# Assessment of Carotid Intraplaque Neovascularization Using Superb Microvascular Imaging in High Risk of Stroke Individuals: Results From a Community-Based Study

**DOI:** 10.3389/fneur.2019.01146

**Published:** 2019-11-07

**Authors:** Ying Wang, Ming Yao, Mi Zou, Shengde Li, Zhitong Ge, Yuehui Hong, Siman Cai, Hongyan Wang, Jianchu Li

**Affiliations:** ^1^Department of Ultrasound, Peking Union Medical College Hospital, Chinese Academy of Medical Sciences and Peking Union Medical College, Beijing, China; ^2^Department of Neurology, Peking Union Medical College Hospital, Chinese Academy of Medical Sciences and Peking Union Medical College, Beijing, China

**Keywords:** carotid plaque, stroke, neovascularization, superb microvascular imaging, ultrasonography, diagnostic imaging

## Abstract

**Background:** Improved stroke risk stratification may improve stroke prevention. We aimed to study the value of a novel Doppler method, superb microvascular imaging (SMI), in correlating plaque thickness and evidence of intra-plaque neovascularization with a history stroke and TIA involving any cerebrovascular territory among community residents considered at high stroke risk.

**Methods:** We selected residents aged at least 40 years from the Donghuashi community in China who had at least three stroke risk factors (including a history of stroke or TIA) and carotid plaque thickness of at least 1.5 mm (but without heavy calcification) and no history of carotid endarterectomy or stenting. In this cross-sectional study, each subject underwent carotid plaque examination with standard ultrasound and SMI. SMI evidence of plaque neovascularization was categorized as none or mild (Grade 1) or moderate or marked (Grade 2) and correlated with past history of stroke or TIA.

**Results:** A total of 131 individuals (mean age 69 ± 8 years, 63% male) met the study inclusion criteria. SMI revealed no or mild neovascularization in 74 subjects (56.5%) and moderate or marked neovascularization in 57 subjects (43.5%). Subjects with moderate or marked neovascularization were more likely to have a history of any territory stroke or TIA, 43.9 vs. 17.6% (*P* = 0.001). Multivariate logistic regression analyses showed a thicker plaque (odds ratio: 2.272, 95% CI: 1.351–3.822, *P* = 0.002) and a history of stroke or TIA (odds ratio: 4.017, 95% CI: 1.719–9.387, *P* = 0.001) significantly correlated with evidence of moderate to marked intra-plaque neovascularization.

**Conclusions:** Moderate to marked intraplaque neovascularization detected by SMI was more likely in subjects with a history of any territory stroke or TIA or thicker plaque. This indicates a potential new role of SMI in stratifying future risk of stroke or other arterial disease complications.

## Introduction

The prevalence of stroke in China has increased over the past decades, leading to high mortality and severe disability among survivors and indicating a heavy disease burden (1, 2). Recent studies confirmed a pronounced association between intraplaque neovascularization and plaque vulnerability in terms of increased risk for neovessel rupture, hemorrhage, and inflammation, which is an evident marker of cerebrovascular disease (3, 4).

Contrast-enhanced ultrasound (CEUS) is largely applied in visualizing intraplaque neovascularization (5–7). CEUS uses microbubbles as a contrast medium or an intravascular tracer. As a result, the use of contrast agents makes CEUS not 100% applicable since allergic reactions may occur in some patients (8). Superb microvascular imaging (SMI; Toshiba Medical Systems, Tokyo, Japan) is a novel Doppler technique. Unlike conventional Doppler imaging, it could provide vascular information by extracting flow signals from either large vessels or smaller microvasculature by advanced filtering algorithms to suppress only background tissue motion without suppressing any slow flow signals. Hence, SMI could detect subtle and slow flow signals to enable the visualization of intraplaque microvascular flow without contrast media (9). Several studies have demonstrated that SMI and CEUS are consistent for the detection of neovascularization in carotid plaques that were verified by histology (10–12). However, little work has been done to demonstrate SMI findings for stroke or TIA. The correlation of neovascularization and plaque thickness was still controversial, and some studies reported that neovascularization was not associated with plaque thickness (13, 14). However, in some other studies, there was an association between neovascularization and plaque thickness (15, 16). The aim of this community-based population study was to correlate the clinical history of stroke or TIA with the plaque thickness and degree of intraplaque neovascularization under the detection of SMI in patients considered at high stroke risk.

## Methods

### Study Sample

Inclusion criteria were

1. Residential dwellers who were continuous residents for at least 6 months and aged at least 40 years of old from the Donghuashi community in China and had
2. At least three stroke risk factors (including a history of stroke or TIA) and had
3. Carotid plaque thickness of at least 1.5 mm but without heavy calcification and had
4. No history of carotid endarterectomy or stenting.

**Figure 1:**
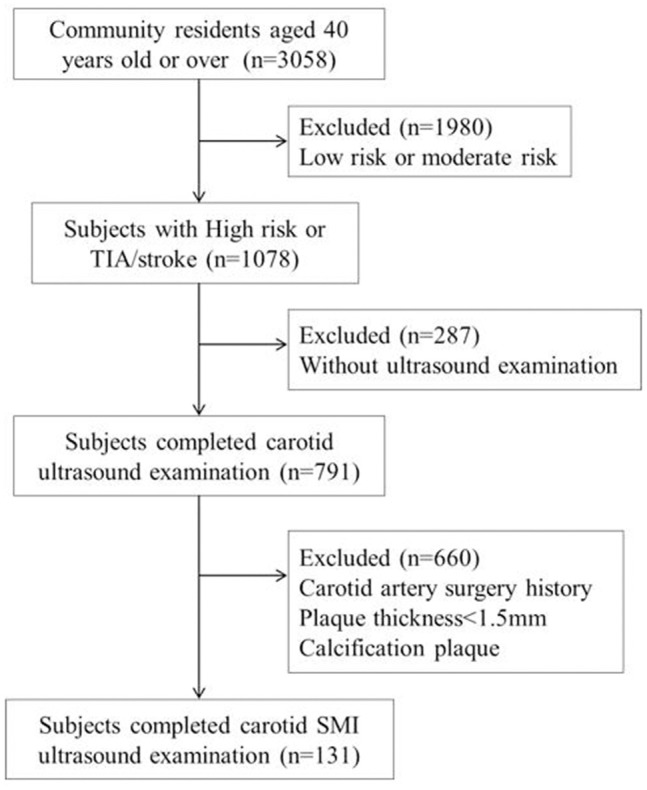
Flow chart of the study sample and a description of the missing data.

Stroke risk factors were evaluated by the information obtained at baseline history, physical examination, 12 lead ECG, and blood tests. Each person was screened for the following eight risk factors: (1) Blood pressure ≥140/90 mmHg. (2) Atrial fibrillation or valvular heart disease; atrial fibrillation was defined as individual with a history of atrial fibrillation diagnosed by clinicians or those were screened by rest 12-lead electrocardiogram. (3) Smoking status; smoking status included being a current smoker and former smoker. Current smokers were defined as those who had smoked within 6 months before the survey. Former smokers were defined as those who used to smoke but had not smoked for at least 6 months at the time of survey. (4) Dyslipidemia (triglyceride ≥2.26 mmol/L, total cholesterol ≥6.22 mmol/L, low-density lipoprotein cholesterol ≥4.14 mmol/L, or high-density lipoprotein cholesterol <1.04 mmol/L). (5) Diabetes, defined according to the 1999 World Health Organization criteria. (6) Lack of exercise, defined as <3 times per week and <30 min each time. (7) Body mass index (BMI) ≥ 26; BMI was calculated as body weight in kilogram divided by body height squared in meters. (8) Family history of stroke. Stroke or TIA was defined as subjects with a medical history of stroke or TIA diagnosed by clinicians, and accurate lesion location was also tried to be collected. The flow chart of the study sample and a description of the missing data are presented in Figure 1.

For these high risk of stroke individuals, getting optimal treatment targets as indicative of good blood pressure, low-density lipoprotein, and glucose level was who kept blood pressure <140/90 mmHg, low-density lipoprotein <2.6, and glycated hemoglobin <7.0%.

The Ethics Committee of the China CSSS, including key neurologists, cardiologists, and epidemiologists, was established to provide ethical approval and technical support to the program. Written informed consent was obtained from each participant.

### Standard Ultrasound Examination

Standard carotid artery ultrasound and SMI were performed using a high-resolution ultrasound system (Aplio 500 UZRI-A500A; Canon Medical Systems, Tokyo, Japan) equipped with a 7.5 MHz linear probe (Aplio PLT-704SBT; Canon Medical Systems). In all participants, both of the common carotid arteries, as well as the internal and external carotid arteries, were examined by radiologists following a standardized protocol using conventional B-mode and color Doppler imaging. Stenosis severity was assessed using the criteria of the Society of Radiologists in Ultrasound. In short, a peak systolic velocity (PSV) of 125 cm/s was considered indicative of 50% stenosis, a lesion and PSV of 125–230 cm/s was considered to indicate 50–69% stenosis, and a lesion and PSV of 230 cm/s was considered to indicate ≥70% stenosis; no detectable patent lumen and no flow on spectral, power, or color Doppler were considered to indicate total occlusion (17).

Carotid plaques were defined as a focal region with a thickness ≥1.5 mm as measured from the media adventitia interface to the lumen–intima interface or as the presence of focal wall thickening that is at least 50% greater than that of the surrounding vessel wall. In cases of individuals with more than one unique plaque, only the thickest plaque was included in our analysis.

### SMI Examination

**Figure 2:**
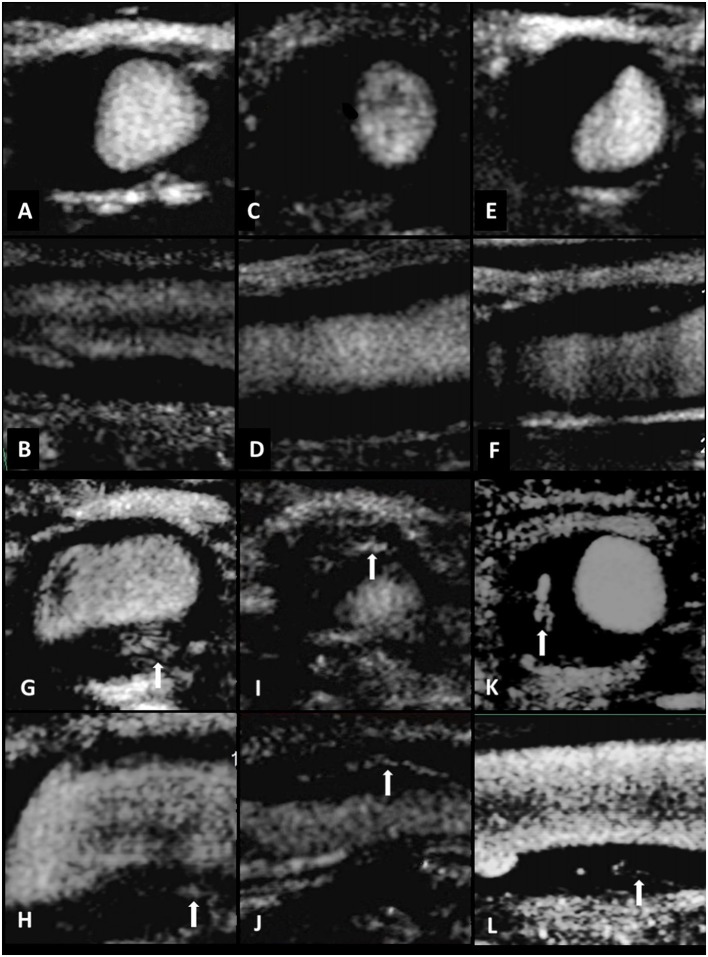
Standard carotid artery plaque ultrasound and SMI in three individuals. Transverse and longitudinal carotid SMI ultrasound of the CCA **(A–F)**. Limited neovascularization was observed within the plaque, suggesting intraplaque vascularization of SMI Grade 1 **(A–F)**. Standard carotid artery plaque ultrasound and SMI in three individuals. Transverse and longitudinal carotid SMI ultrasound of the CCA **(G–L)**. Multiple short-line or strip-like hyperintense echo could be observed both in transverse and longitudinal direction. The arrows indicate moderate or marked neovascularization within the plaque, indicating intraplaque vascularization of SMI Grade 2 **(G–L)**.

When the carotid plaque was observed, we then conducted SMI to investigate the flow signals in the plaque with the probe in the same position. The region of interest was positioned to include the entire plaque. Other settings were a mechanical index of 1.5, a frame rate of 50–60 fps, a velocity range of 2.0 cm/s, and a dynamic range of 55–60 dB. The patient was instructed to continue to breathe calmly. The plaque was visualized and the video images were stored on the hard disk of the ultrasound system. After the scan was finished, the video was played back and was observed whether there were new blood vessels in the plaque. Calcification in mix plaques in SMI mode shows similar signs that could be interpreted as neovascularization. However, it can distinguish between them. New vessels will flow with time, but the development of calcification is static, brighter than new blood vessels, and can coincide with two-dimensional images. Therefore, it can eliminate the impact of calcification on the observation of neovascularization (11). Neovascularization was identified by the short-line or strip-like hyperintense echo. The blood vessels in the plaque were categorized as follows: Grade 1 = no or mild blood flow signal in the plaque (Figure 2); Grade 2 = moderate or marked blood flow signal in the plaque.

All carotid artery scans were performed by four researchers (YW, with 4 years of experience with US; MZ, with 3 years of experience with US; ZTG, with 3 years of US; and SMC with 3 years of experience with US). All SMI scans were performed by two researchers (LZ and WLF, with more than 10 years of experience with US). All of the researchers were blinded to the participant histories.

The images were graded by two independent radiologists (YW and LZ) who had 4 and 11 years of experience in US. If any disagreement occurred, a third senior radiologist (HYW) was consulted who had more than 10 years of experience in carotid ultrasonography until a consensus was reached. All three radiologists were blinded to the participant histories.

### Statistical Analysis

Quantitative data are presented as the means ± standard deviation (SD). Qualitative data are presented as frequencies. The chi-squared test or Fisher's exact test was applied to categorical variables, while an independent *t*-test was used to compare continuous variables. The correlation between intraplaque neovascularization on SMI and the risk factors we tested, including hypertension, atrial fibrillation or valvular heart disease, smoking including current smoker and former smoker, dyslipidemia, diabetes, lack of exercise, BMI, and family history of stroke, was analyzed. Multivariate logistic regression analyses were performed for all factors correlated with SMI in univariate analyses (male, plaque thickness, and previous stroke or TIA). Receiver operating characteristic (ROC) curve analyses were used to calculate the area under the curve (AUC) for the plaque thickness to determine the best cutoff value for identifying moderate to marked intraplaque neovascularization on SMI. Sensitivity and specificity were also calculated. *P* < 0.05 was considered significant in all tests. The kappa value was calculated to assess interobserver variability. The statistical analyses were performed using SPSS (Version 19.0, SPSS Chicago, IL, USA) software.

## Results

### Study Sample

As shown in Table 1 among the 131 subjects, only some had what we considered to be optimal control of lifestyle risk factors, like non-smoking (55.7%) and with adequate physical activity (61.1%). Medication usage included antihypertensive medication (66.4%), statin (47.2%), diabetes medication (42%), and anti-thrombotic medications (5.8%). The proportion of subjects achieving their treatment targets was 68.7% for low-density lipoprotein, 66.4% for blood pressure, and 68.7% for diabetes. Previous stroke or TIA involving any cerebrovascular territory occurred in 38 (29%) subjects. For 25 individuals with previous stroke, 16 of them could not tell the accurate lesion location for incomplete data, and among nine individuals whose data of stroke lesion location were accurately recorded, eight of them suffered ipsilateral stroke with the plaque side and 1 participant had contralateral stroke. Among 131 subjects, 77 (58.8%), 34 (26%), and 20 (15.3%) individuals had <50%, 50–69, and ≥70% carotid stenosis, respectively.

**Table 1:** Baseline characteristics of all consecutive patients (*n* = 131).

**Risk factors**	
Age, years	69.56 ± 7.99
Male gender	83 (63.4)
BMI, kg/m^2^	25.97 ± 3.59
Diabetes mellitus	69 (52.7)
Hypertension	112 (85.5)
Atrial fibrillation	9 (6.9)
Current or former smoker	58 (44.3)
Current smoker	48 (36.6)
Lack of exercise	51 (38.9)
Lipids, mmol/L	
LDL	2.41 ± 0.74
HDL	1.52 ± 0.35
Total cholesterol	4.63 ± 1.01
Triglycerides	1.60 ± 0.54
Use of drugs	
Antihypertensive medication	87 (66.4)
Statins	62 (47.3)
Diabetes medication	55 (42)
Anti-thrombotic medications	5 (3.8)
Clinical history	
Coronary artery disease	30 (22.9)
MI	3 (2.3)
TIA or stroke	38 (29)
^*^Ipsilateral stroke	8 (88.9)

*Data are expressed as the mean ± SD or No. (percentage) of subjects*.

*BMI, indicates body mass index; LDL, low-density lipoprotein; HDL, high-density lipoprotein; TIA, transient ischemic attack*.

* *Means data were incomplete*.

SMI revealed no or mild intraplaque neovascularization (Grade 1) in 74 subjects (56.5%), and moderate to marked intraplaque neovascularization (Grade 2) was noted in 57 subjects (43.5%), as shown in Table 2.

**Table 2:** Plaque characterization by ultrasonography.

**Carotid ultrasound findings**	
Multiple plaques	115 (87.8)
Max plaque thickness, mm	2.60 ± 0.81
Stenosis <50%	77 (58.8)
Stenosis 50–69%	34 (26)
Stenosis ≥70%	20 (15.3)
Microvascular flow signal in SMI	
Grade 1	74 (56.5)
Grade 2	57 (43.5)

*SMI indicates superb microvascular imaging*.

### Factors Associated With the Presence of Neovascularization

As shown in Table 3, compared with subjects with SMI evidence of no or mild neovascularization, subjects with moderate or marked intraplaque neovascularization were more likely to be male, 73.7 vs. 55.4% (*P* = 0.024), and they had thicker plaques (2.50 ± 0.70 vs. 2.89 ± 0.77 mm, *P* = 0.001) and more likely to be with a history of stroke or TIA event, 43.9 vs. 17.6% (*P* = 0.001).

**Table 3:** Intraplaque neovascularization on SMI and its association with clinical characteristics.

	**Intraplaque neovascularization**		
	**Grade 1 (*n* = 74)**	**Grade 2 (*n* = 57)**	***P*-value**
Risk factors			
Age, years	69.19 ± 8.24	70.04 ± 7.69	0.55
Male gender	41 (55.4)	42 (73.7)	**0.024**
BMI, kg/m^2^	26.00 ± 3.51	25.94 ± 3.72	0.918
Diabetes mellitus	38 (51.4)	31 (54.4)	0.433
Hypertension	63 (85.1)	49 (86)	0.549
Atrial fibrillation or valvular heart disease	4 (5.4)	5 (8.8)	0.339
Current or former smoker	30 (40.5)	28 (49.1)	0.211
Current smoker	26 (35.1)	22 (38.6)	0.410
Overweight or obese	22 (29.7)	14 (24.6)	0.324
Lack of exercise	30 (40.5)	21 (36.8)	0.402
Family history of stroke	19 (25.7)	19 (33.3)	0.222
Lipids, mmol/L			
LDL	2.40 ± 0.73	2.47 ± 0.76	0.448
HDL	1.52 ± 0.37	1.53 ± 0.33	0.849
Total cholesterol	4.60 ± 0.98	4.67 ± 1.07	0.718
Triglycerides	1.61 ± 0.50	1.58 ± 0.58	0.801
Multiple plaques	63 (85.1)	52 (91.2)	0.217
Max plaque thickness, mm	2.50 ± 0.70	2.89 ± 0.77	**0.001**
Clinical history			
Coronary artery disease	17 (23)	13 (22.8)	0.576
MI	1 (1.4)	2 (3.5)	0.402
TIA or stroke	13 (17.6)	25 (43.9)	**0.001**

*Data are expressed as the mean ± SD or No. (percentage) of subjects*.

*The p values are provided in the table with bold values representing significant P < 0.05*.

The ROC curve demonstrated that a value of plaque thickness of 2.25 mm was the best cutoff to predict SMI evidence of moderate to marked neovascularzation [AUC = 0.672 (95% CI: 0.579–0.765, *P* < 0.01); sensitivity = 77.2%; specificity = 51.4%].

The presence of moderate or marked intraplaque neovascularization on SMI among subjects with documented plaques was not associated with any other cerebrovascular risk factors we tested, including hypertension, atrial fibrillation or valvular heart disease, smoking including current smoker and former smoker, dyslipidemia, diabetes, lack of exercise, BMI, and family history of stroke.

### Multivariate Analysis for Detection of Moderate or Marked Intraplaque Neovascularization

As shown in Table 4, multivariate logistic regression analyses of cerebrovascular risk factors and carotid ultrasound data showed that thicker plaque (odds ratio: 2.272, 95% CI: 1.351–3.822, *P* = 0.002) and a history of stroke or TIA event (odds ratio: 4.017, 95% CI: 1.719–9.387, *P* = 0.001) were significantly associated with individuals with moderate to marked intraplaque neovascularization, compared with subjects with no or mild neovascularization.

**Table 4:** Multivariate analysis for patients with moderate or marked intraplaque neovascularization.

	**Moderate or marked intraplaque neovascularization**	
	**Odds ratio (95% CI)**	***P*-value**
Multivariate analysis		
Male gender	0.544 (0.242–1.226)	0.142
Max plaque thickness, mm	2.272 (1.351–3.822)	**0.002**
Previous stroke/TIA	4.017 (1.719–9.387)	**0.001**

*CI indicates confidence interval*.

*The p values are provided in the table with bold values representing significant P < 0.05*.

### Reproducibility of SMI Findings

To establish the reproducibility of our qualitative assessment, intraobserver and interobserver agreement was determined by applying Cohen's kappa statistic to the intraplaque neovascularization grading by two different readers and by one reader at an interval of more than 7 days using video loops of SMI. Intraobserver and interobserver agreement for kappa coefficient was good, 0.83 (95% CI: 0.79–0.87) and 0.77 (95% CI: 0.73–0.80), respectively.

## Discussion

In the present study, we confirmed the hypothesis that intraplaque neovascularization identified using SMI is more likely in persons with a history of stroke or TIA. We also showed that SMI evidence of moderate to marked neovascularzation was more common in thicker plaques. The relationship of plaque thickness for predicting SMI evidence of moderate to marked neovascularization was further analyzed. The result indicated that a value of plaque thickness of 2.25 mm was the best cutoff value for predicting SMI evidence of moderate to marked neovascularzation. To the best of our knowledge (10–12, 18), this is the first study in which SMI findings of intraplaque neovascularization were correlated to the subjects' clinical histories, and also the study with the largest sample for neovascularization detection by SMI until now. It has been shown that intraplaque neovascularization is a frequently studied carotid plaque feature since it has been described to be associated with plaque instability (19–22). Plaque instability, which leads to plaque rupture and clinical events, may be triggered by the disruption and leakage of immature neovessels originating from the adventitial vasa vasorum. Newly formed microvessels contain newly formed and frequently insufficient endothelium. It could lead to microvascular leakage and thereby potentially result in plaque hemorrhage and progression. The neovasculature growth into the plaque and increased endothelial permeability are associated with plaque inflammation, so plaque enhancement has been recognized as a sign of plaque inflammation (23–25). Van der Donckt et al. found that the atherosclerotic plaques of ApoE KO Fbn1C1039G^+/−^ mice contained highly leaky plaque neovessels, resulting in plaque rupture, myocardial infarction, stroke, and sudden death (26).

Recent studies have shown that CEUS is of great value in the detection and assessment of neovascularization in plaques. The study by Li showed that 72% of patients had intraplaque neovascularization confirmed by CEUS in the carotid atherosclerosis population. In a previous study using CEUS, Daniel et al. found that carotid plaque neovascularization was significantly associated with future cardiovascular events (myocardial infarction and stroke or TIA) with an ~4-fold increase in risk (27). A study by Li also found that carotid artery plaques in stroke patients had significantly more angiogenesis than patients in the control group (87 vs. 54%, *P* < 0.001) (28). However, the operation of CEUS is complex and requires the support of contrast agents and nurses. Therefore, it cannot be used as a screening tool for finding unstable plaques. Thus, developing a non-invasive imaging method to assess plaque vulnerability on the basis of plaque vascularization is highly relevant.

SMI is a novel technique for detecting blood flow in micro-vessels, enabling the detection of micro-vessels at a much lower speed. Recent validation studies revealed a positive direct correlation between SMI, CEUS, and other imaging modalities and quantitative histology. Oura et al. observed intraplaque enhancement using CEUS and SMI in 19 patients, and found 100% specificity and 63% sensitivity for neovascularization using SMI (10). Zhang et al. detected neovascularization in 39 patients with severe carotid artery stenosis (64 carotid plaques) and found 65% specificity and 100% sensitivity for detection of neovascularization using SMI. The good consistency of SMI and CEUS grading was shown (κ = 0.860 > 0), and the clinical symptoms and severity were positively correlated with SMI grading (rs = 0.592 > 0) (11). A study by Hoshino et al. compared SMI and MRI in detecting carotid plaque neovascularization and found microvascular detected by SMI was identified as a significant predictor of intraplaque hemorrhage, as evaluated by MRI (OR: 8.46; *P* = 0.018) (12).

Consistent with the previous studies, in our study, nearly half of the individuals enrolled in the study with carotid plaques had moderate or marked intraplaque neovascularization using SMI (43.5%). In our present study, the plaques were thicker in the Grade 2 group than in the Grade 1 group, suggesting that plaque thickness was positively related with intraplaque neovascularization detected by SMI, which was consistent with previous studies (15, 16). Plaque thickness was an important factor for stroke as it is positive relative to the artery stenosis, which is a very important indicator for stroke monitoring (16). After adjustment for plaque thickness, intraplaque neovascularization detected by SMI remained to be significantly associated with previous stroke or TIA, demonstrating that moderate or marked intraplaque neovascularization was significantly associated with previous stroke or TIA. These findings, based on credible evidence, support the concept that increased intraplaque neovascularization detected by SMI is emblematic of the inflamed arterial vessels associated with plaque instability and is more commonly found in persons with previous stroke or TIA.

Our study showed that plaque neovascularization on ultrasound was significantly associated with previous stroke or TIA in any cerebrovascular territory. Whether this may be a useful instrument predicting the risk of ipsilateral stroke needs to be verified in future prospective observational studies, preferably in the context of current optimal medical intervention for arterial disease risk factors. Current optimal medical treatment consists of risk factor definition and identification, lifestyle modification, and appropriate use of medication. In the current study, the medical treatment being used is not very good, with much room for improvement in terms of getting people to their treatment targets. Carotid surgery or stenting for asymptomatic severe carotid stenosis is considered in routine clinical practice (29–31). However, recent advances in medical therapy have been associated with a reduced risk of stroke to a level that is similar to or lower than that observed in previous randomized trials of carotid surgery (32–35). Medical management probably also has a strong influence on the detection of imaging evidence of neovascularization. Population screening using imaging methods such as SMI may be helpful in constructing improved stroke risk stratification methods. This needs to be confirmed in future prospective observational studies in the context of current optimal medical intervention. If successful in stroke risk stratification, particularly high-risk patients may then be better selected for additional medical interventions or appropriate carotid artery procedures.

Our study has some strength. First, we initially investigated the determinants of subclinical atherosclerosis and plaque burden in a community-based population. With the increasing incidence of stroke in China and worldwide, such studies are urgently needed. Second, this is the study with the biggest sample for detection neovascularization by using SMI. Third, we used a semiquantitative visual approach to evaluate intraplaque neovascularization, and categorized two gradings of plaque neovascularization in SMI mode. That's because SMI is not as sensitive as CEUS (10, 11) and could be interfered by factors such as plaque calcification; thus, SMI diagnostic performance is more solid for severe neovascularization detection rather than small and tiny neovascularization. These two SMI grading categories are more accurate for neovascularization observation and are better mastered by doctors.

There were some limitations to this study. First, we did not compare the characteristics of those excluded in the screening process (identified as non-high risk of stroke) with those included (identified as high risk of stroke) in our study due to lack of data, so our results can only apply to those at high risk of stroke. Second, although previous studies have validated SMI against CEUS, this has not been done in this study. Third, we did not analyze the ultrasound characteristics such as plaque echogenicity and surface, and the data for previous stroke lesion location were partially absent; new studies would be designed for these. Fourth, for lack of complete data, we could not differentiate brain vascular events ipsilateral to the index carotid plaques from events in any cerebrovascular territory.

## Conclusions

Moderate to marked intraplaque neovascularization on SMI was more likely in persons with a history of stroke or TIA and with larger plaques. Our findings support the concept that intraplaque neovascularization is associated with plaque instability and vulnerability. Therefore, the use of SMI may provide a non-invasive adjunctive “window” to identify “vulnerable” plaques and may serve as a valuable screening tool to identify patients at high risk of future stroke or TIA or other complications of arterial disease. Further study is recommended using prospective risk stratification cohort studies in the context of current optimal medical intervention (lifestyle coaching and medications).

## Data Availability Statement

The datasets generated for this study are available on request to the corresponding author.

## Ethics Statement

The studies involving human participants were reviewed and approved by The Ethics Committee of the China CSSS, including key neurologists, cardiologists, and epidemiologists, was established to provide ethical approval and technical support to the program. The patients/participants provided their written informed consent to participate in this study.

## Author Contributions

YW, MZ, ZG, and SC performed the general ultrasound examination and collected the clinical data. YW wrote the manuscript. MY, SL, and YH evaluated the cerebrovascular disease and made the diagnosis of previous stroke and TIA. HW and JL designed the whole experiments and supervised the whole project. All authors listed in the manuscript are the guarantors of this work and, as such, had full access to all the data used in the study and take responsibility for the integrity of the data and the accuracy of the data analysis.

### Conflict of Interest

The authors declare that the research was conducted in the absence of any commercial or financial relationships that could be construed as a potential conflict of interest.
